# The causal association between thyroid disease and gout: A Mendelian randomization study

**DOI:** 10.1097/MD.0000000000035817

**Published:** 2023-11-03

**Authors:** Shuai Song, Congcong Jia, ChunJing Li, Yuxia Ma

**Affiliations:** a Department of Traditional Chinese Medicine External Treatment Center, Affiliated Hospital of Shandong University of Traditional Chinese Medicine, Jinan, Shandong, China; b Department of Nephrology, Shandong First Medical University Affiliated Occupational Disease Hospital, Jinan, Shandong, China; c College of Acupuncture and Tuina, Shandong University of Traditional Chinese Medicine, Jinan, Shandong, China.

**Keywords:** autoimmune hyperthyroidism, autoimmune hypothyroidism, gout, Mendelian randomization, thyroid cancer, thyroid nodules

## Abstract

Observational studies have reported some associations between thyroid disease and gout, but the causal relationship between the 2 is not clear. We used Mendelian randomization (MR) Analysis to investigate the causal association between some thyroid diseases (autoimmune hypothyroidism, autoimmune hyperthyroidism, thyroid nodules, and thyroid cancer) and gout. GWAS data were used for analysis. The exposure factors were autoimmune hypothyroidism, autoimmune hyperthyroidism, thyroid nodules and thyroid cancer, and the outcome variables were gout. IVW, MR-Egger, Weighted median and Weighted mode were used for MR analysis. Cochran Q test MR-PRESSO and MR-Egger intercept analysis were used to detect heterogeneity and multi directivity. Autoimmune hypothyroidism has a causal effect on gout, IVW results show (OR = 1.13, 95% CI = 1.03–1.21, P_FDR_ = 0.0336); Autoimmune hyperthyroidism has a causal effect on gout, IVW results show (OR = 1.07, 95% CI = 1.01–1.12, P_FDR_ = 0.0314); Thyroid cancer has no causal effect on gout, IVW results show (OR = 1.03, 95% CI = 0.98–1.09, P_FDR_ = 0.297); Thyroid nodules has no causal effect on gout, IVW results show (OR = 1.03, 95% CI = 0.98–1.08, P_FDR_ = 0.225); Reverse MR Studies show that gout have no causal effect on the above thyroid diseases. Autoimmune hypothyroidism and autoimmune hyperthyroidism increase the risk of gout.

## 1. Introduction

Thyroid disease is a common clinical disease, and its prevalence increases with age.^[1]^ Common clinical thyroid diseases include autoimmune thyroid disease, thyroid nodules and thyroid cancer.^[2]^ The prevalence of autoimmune thyroid dysfunction (autoimmune hypothyroidism and autoimmune hyperthyroidism) in the general population is 5%.^[3]^ Epidemiological studies have found that Hashimoto thyroiditis is a common cause of hypothyroidism.^[4]^ The global prevalence of hyperthyroidism is 0.2% to 1.3%, and Graves is the main cause.^[5]^ In addition, thyroid nodules and thyroid cancer also have a high incidence in the general population, adding a serious economic burden to society.^[6,7]^ Gout is a metabolic disease caused by increased uric acid concentrations and deposits of sodium urate crystals, affecting approximately 410,000 adults worldwide.^[8,9]^ Gout seriously affects People Daily physiological function and quality of life, and brings high medical costs and heavy mental burden to patients. In recent years, the relationship between thyroid disease and gout has attracted wide attention. Studies have shown that many thyroid diseases are associated with an increased risk of gout.^[10,11]^ However, some studies have shown the opposite.^[12]^ Whether there is a link between gout and thyroid disease is controversial. However, the traditional observational studies usually have confounders and reverse causality, and there are some limitations in the causal inference of association. Therefore, no studies have proven a causal relationship between thyroid disease and gout.

Mendelian randomization (MR) is a genetic epidemiological research method based on Mendelian laws of inheritance, using single nucleotide polymorphic sites (SNPs) as instrumental variables (IVs). The potential causal relationship between exposure factors and outcomes can be inferred.^[13,14]^ Because genes are randomly assigned at the time of conception, genetic variation precedes disease development and is unaffected by common confounding factors such as environment, socioeconomic status, and behavioral factors, therefore, MR Research has a reasonable time sequence when inferring causal association, avoiding confounding bias and reverse causal association, and making the research results more close to the real situation.^[15,16]^

Genome-wide association Studies (GWAS) publicly publish pooled data on hundreds of thousands of exposures and disease associations with genetic variation, these GWAS data allow researchers to estimate genetic associations in large samples of data. Therefore, this study adopted the 2-sample MR Study method to explore the potential causal relationship between some thyroid diseases (autoimmune hypothyroidism, autoimmune hyperthyroidism, thyroid nodules and thyroid cancer) and gout, providing theoretical basis for the treatment and prevention of gout.

In this MR Study, autoimmune hypothyroidism, autoimmune hyperthyroidism, thyroid nodules and thyroid cancer were used as exposure factors, and single nucleotide polymorphisms (SNPs) significantly associated with exposure factors were used as IVs, the outcome variable was gout. This MR Study should be based on 3 basic assumptions^[17]^: IVs must be closely related to exposure factors (autoimmune hypothyroidism, autoimmune hyperthyroidism, thyroid nodules, and thyroid cancer); IVs cannot be associated with any possible confounding factors; and IVs can only affect outcome factors (gout) through exposure factors.

## 2. Methods

### 2.1. Source of data

All datasets in this MR Study are from IEU OpenGWAS project (https://gwas.mrcieu.ac.uk). Autoimmunity hypothyroidism GWAS included 26,342 patients and 59,827 controls of European descent. Autoimmunity hyperthyroidism GWAS included 962 patients and 172,976 controls of European descent. Thyroid nodule GWAS included 455 patients and 218,337 controls of European descent. Thyroid cancer GWAS included 989 patients and 174,006 controls of European descent. Gout GWAS included 3576 patients and 147,221 controls of European descent. The MR Study is based on publicly available GWAS data, Therefore, there is no need to seek patient consent and ethics committee approval (Table 1).

**Table 1:** The data for the description of the contributing study were obtained from the IEU OpenGWAS project.

Trait	Hypothyroidism	Autoimmune hyperthyroidism	Benign neoplasm of thyroid gland	Malignant neoplasm of thyroid gland (all cancers excluded)	Gout
nCases/nControls	26,342/59,827	962/172,976	455/218,337	989/174,006	3576/147,221
Number of SNPs	16,378,441	16,380,189	16,380,466	16,380,316	16,380,152
Population	European	European	European	European	European
Sex	Males and females	Males and females	Males and females	Males and females	Males and females
Release date	2021	2021	2021	2021	2021
Access address	https://gwas.mrcieu.ac.uk/datasets/finn-b-HYPOTHYROIDISM/	https://gwas.mrcieu.ac.uk/datasets/finn-b-AUTOIMMUNE_HYPERTHYROIDISM/	https://gwas.mrcieu.ac.uk/datasets/finn-b-CD2_BENIGN_THYROID/	https://gwas.mrcieu.ac.uk/datasets/finn-b-C3_THYROID_GLAND_EXALLC/	https://gwas.mrcieu.ac.uk/datasets/finn-b-M13_GOUT/

SNPs = single nucleotide polymorphic sites.

### 2.2. Instrumental variable selection

First, when independent SNPs associated with autoimmune hyperthyroidism and autoimmune hypothyroidism were selected, *P* < 5 × 10-8. However, when selecting independent SNPs associated with thyroid cancer and thyroid nodules, due to the small number of SNPs, we expanded the threshold to *P* < 5 × 10-6^.[18,19]^Secondly, SNPs significantly associated with gout were screened (*P* < 5 × 10-8). Third, we assessed the relationship between SNPS strongly associated with exposure and linked disequilibrium (LD) SNPS associated with possible known confounders. Then, remove the tool variable for the link unbalance (LD) state (r^2^ < 0.001, aggregation distance > 10,000kb).Fourth, we eliminated allele SNPS with frequencies <1%. Fifth, we further eliminate the interference of confounding factors through PhenoScanncer database. Finally, the F statistic is calculated to evaluate whether there is weak instrumental variable offset in the selected instrumental variable. F > 10 indicates that there is no weak instrumental variable offset, so as to further verify the MR Correlation hypothesis. After calculation, the F statistics of relevant IV in this MR Study are all >20, suggesting that it is not susceptible to the bias of weak IVs. The extracted IVs were found to correspond to SNPs in the outcome database, and then the consistency of effect genes was coordinated to exclude palindromic sequences (SNPS composed of alleles and their complementary bases).

### 2.3. Statistical analysis

This study mainly adopts inverse variance weighting method(IVW). The results of IVW are the main indicators of this study. Cochran Q was used to test heterogeneity. The fixed effects model is used when there is no heterogeneity, and the random effects model is used when there is heterogeneity. In addition, methods of MR-Egger, weighted median, and weighted mode are used to ensure the robustness of the analysis results. The intercept term of MR-Egger regression (compared with 0, the intercept has no statistical significance, indicating that there is no horizontal pleiotropy in this study) and MR-PRESSO were used to test whether there is horizontal pleiotropy in snp. We adopted the leave-one-out method for sensitivity analysis. After 1 SNP was excluded each time, the remaining SNPS were used as gene IVs for IVW effect analysis again, so as to determine the influence of individual SNPS on the analysis results and ensure the stability of MR results. There were 4 exposure factors in this study. FDR method was used for multiple correction of associated *P* values, and P_FDR_ < 0.05 was considered statistically significant. All statistical analyses were performed using R (version 4.2.4).

## 3. Results

### 3.1. Causal association by Mendelian randomization analysis

#### 3.1.1. Impact of autoimmune hypothyroidism on gout.

**Figure 1.**
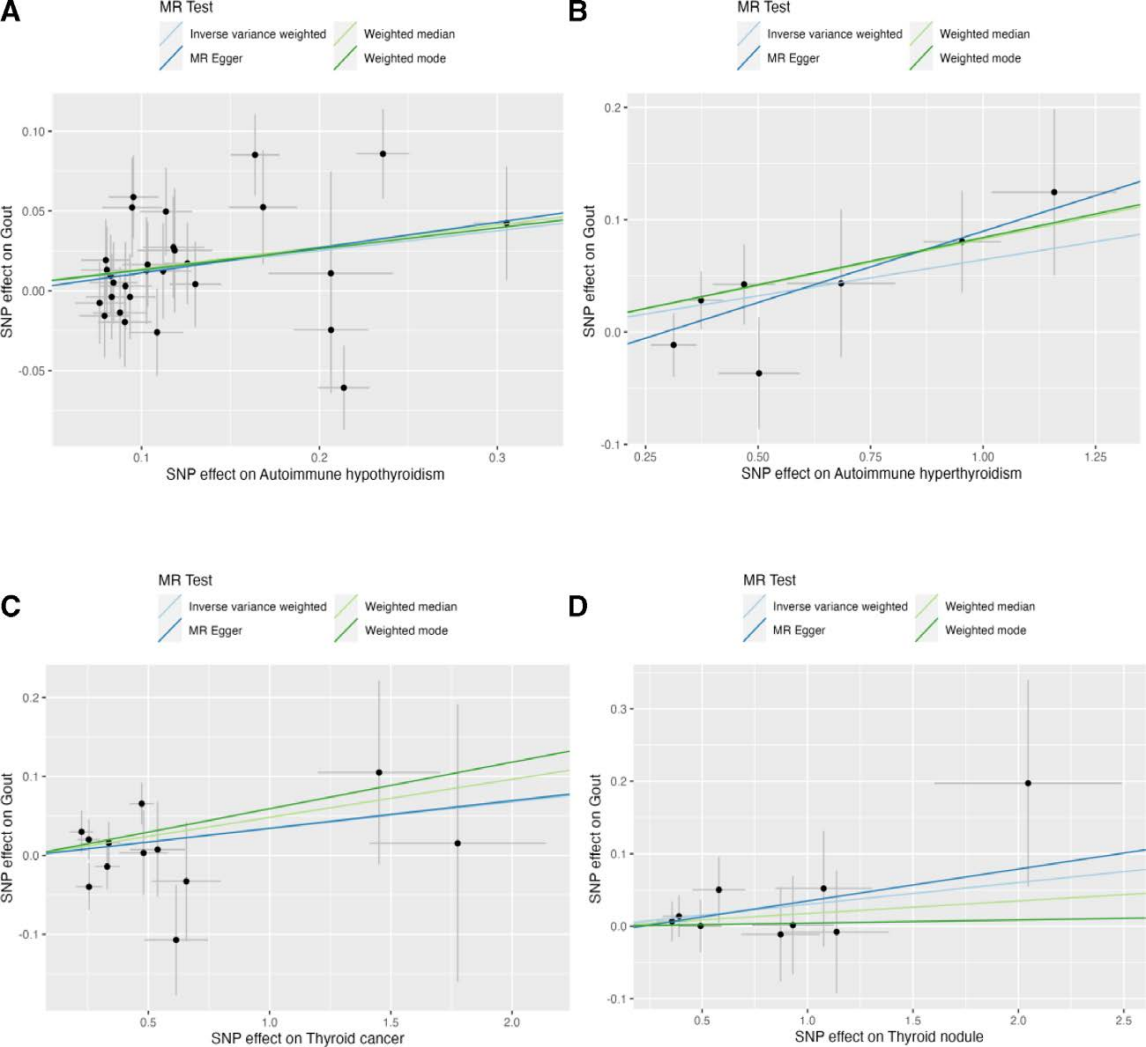
Plots of MR estimates of the causal relationship between thyroid disease and gout with 4 methods (IVW approach, MR-Egger, weighted median, weighted mode). (A) The scatterplot of the causal effect of autoimmune hypothyroidism on gout; (B) the scatterplot of the causal effect of autoimmune hypethyroidism on gout; (C) the scatterplot of the causal effect of thyroid cancer on gout; (D) the scatterplot of the causal effect of thyroid nodule on gout.

29 SNPs associated with autoimmune hypothyroidism were extracted as IVs. The results of MR Analysis shown that, IVW (OR = 1.13, 95% CI = 1.03–1.24, P_FDR_ = 0.0336), MR-Egger (OR = 1.17, 95% CI = 0.92–1.49, P_FDR_ = 0.42), Weighted median (OR = 1.15, 95% CI = 1.00–1.31, P_FDR_ = 0.10), and Weighted mode (OR = 1.14, 95% CI = 0.89–1.47, P_FDR_ = 0.41) (Fig. 1A). The results of MR-Egger regression (Intercept = −0.004, *P* = .77) and MR-PRESSO (*P* = .128) showed that there was no horizontal pleiotropy. Tested by Cochran Q, heterogeneity was not observed (*P* value >.05).Sensitivity analyses with the leave-one-out method suggested that the MR analysis results were reliable (Supplementary Figure 1A, http://links.lww.com/MD/K489). The direction of MR-Egger, Weighted median and Weighted mode were consistent with the IVW results, therefore, IVW results can be considered as the main reference index.

#### 3.1.2. Impact of autoimmune hyperthyroidism on gout.

7 SNPs associated with autoimmune hyperthyroidism were extracted as IVs. The results of MR Analysis show that, IVW (OR = 1.07, 95% CI = 1.01–1.12,P_FDR_ = 0.0314), MR-Egger (OR = 1.13, 95% CI = 1.00–1.28, P_FDR_ = 0.40), Weighted median (OR = 1.09, 95% CI = 1.02–1.16, P_FDR_ = 0.04) and Weighted mode (OR = 1.09, 95% CI = 1.01–1.18, P_FDR_ = 0.32) (Fig. 1B). The results of MR-Egger regression (Intercept = −0.04, *P* = .32) and MR-PRESSO (*P* = .158) showed that there was no horizontal pleiotropy. Tested by Cochran Q, heterogeneity was not observed (*P* value>.05). Sensitivity analyses with the leave-one-out method suggested that the MR analysis results were reliable (Supplementary Figure 1B, http://links.lww.com/MD/K489). The direction of MR-Egger, Weighted median and Weighted mode were consistent with the IVW results, therefore, IVW results can be considered as the main reference index.

#### 3.1.3. Impact of thyroid cancer on gout.

12 SNPs associated with thyroid cancer were extracted as IVs. The results of MR Analysis show that, IVW (OR = 1.03, 95% CI = 0.98–1.09, P_FDR_ = 0.297), MR-Egger (OR = 1.04, 95% CI = 0.91–1.18, P_FDR_ = 0.61), Weighted median (OR = 1.05, 95% CI = 0.97–1.13, P_FDR_ = 0.29), and Weighted mode (OR = 1.06, 95% CI = 0.95–1.18, P_FDR_ = 0.60) (Fig. 1C). The results of MR-Egger regression (Intercept = −0.0004, *P* = .99) and MR-PRESSO (*P* = .321) showed that there was no horizontal pleiotropy. Tested by Cochran Q, heterogeneity was not observed (*P* value>.05). Sensitivity analyses with the leave-one-out method suggested that the MR analysis results were reliable (Supplementary Figure 1C, http://links.lww.com/MD/K489).

#### 3.1.4. Impact of thyroid nodule on gout.

9 SNPs associated with thyroid nodule were extracted as IVs. The results of MR Analysis show that, IVW (OR = 1.03, 95% CI = 0.98–1.08, P_FDR_ = 0.225), MR-Egger (OR = 1.05, 95% CI = 0.94–1.16, P_FDR_ = 0.59), Weighted median (OR = 1.02, 95% CI = 0.95–1.08, P_FDR_ = 0.59), and Weighted mode (OR = 1.00, 95% CI = 0.91–1.11, P_FDR_ = 0.93) (Fig. 1D). The results of MR-Egger regression (Intercept = −0.009, *P* = .78) and MR-PRESSO (*P* = .968) showed that there was no horizontal pleiotropy. Tested by Cochran Q, heterogeneity was not observed (*P* value>.05). Sensitivity analyses with the leave-one-out method suggested that the MR analysis results were reliable (Supplementary Figure 1D, http://links.lww.com/MD/K489).

The results of MR Analysis are shown in Table 2.

**Table 2:** Main results of Mendelian randomization analysis.

Exposure	N SNPs	Inverse variance weighted			MR-egger				Weighted median		Weighted mode		MP-PRESSO
		P _FDR_	OR (95% CI)	Q statistics (*P* value)	P _FDR_	OR (95% CI)	Q statistics (*P* value)	Intercept (*P* value)	P _FDR_	OR (95% CI)	P _FDR_	OR (95% CI)	*P* value
Autoimmune Hypothyroidism	29	0.0336	1.13 (1.03–1.24)	1.11	0.42	1.17 (0.92–1.49)	.09	.77	0.10	1.15 (1.00–1.31)	0.41	1.14 (0.89–1.47)	.128
Autoimmune Hyperthyroidism	7	0.0314	1.07 (1.01–1.12)	.68	0.40	1.13 (1.00–1.28)	.74	.32	0.04	1.09 (1.02–1.16)	0.32	1.09 (1.01–1.18)	.158
Thyroid cancer	12	0.297	1.03 (0.98–1.09)	.36	0.61	1.04 (0.91–1.18)	.28	.99	0.29	1.05 (0.97–1.13)	0.60	1.06 (0.95–1.18)	.321
Thyroid nodule	9	0.225	1.03 (0.98–1.08)	.97	0.59	1.05 (0.94–1.16)	.94	.78	0.59	1.02 (0.95–1.08)	0.93	1.00 (0.91–1.11)	.968

### 3.2. Impact of gout on autoimmune hypothyroidism, autoimmune hyperthyroidism, thyroid cancer and thyroid nodule

#### 3.2.1. Impact of gout on autoimmune hypothyroidism.

7 SNPs associated with gout were extracted as IVs. The results of MR Analysis show that, IVW (OR = 0.99, 95% CI = 0.92–1.07, *P* = .78), MR-Egger (OR = 0.94, 95% CI = 0.81–1.10, *P* = .47), Weighted median (OR = 1.03, 95% CI = 0.97–1.08, *P* = .33), and Weighted mode (OR = 1.02, 95% CI = 0.96–1.09, *P* = .49).

#### 3.2.2. Impact of gout on autoimmune hyperthyroidism.

7 SNPs associated with gout were extracted as IVs. The results of MR Analysis show that, IVW (OR = 0.97, 95% CI = 0.83–1.13, *P* = .68), MR-Egger (OR = 1.06, 95% CI = 0.77–1.45, *P* = .74), Weighted median (OR = 0.95, 95% CI = 0.79–1.15, *P* = .62), and Weighted mode (OR = 0.92, 95% CI = 0.68–1.23, *P* = .57).

#### 3.2.3. Impact of gout on thyroid nodule

7 SNPs associated with gout were extracted as IVs. The results of MR Analysis show that, IVW (OR = 1.02 95% CI = 0.84–1.24, *P* = .85), MR-Egger (OR = 0.94, 95% CI = 0.64–1.38, *P* = .77), Weighted median (OR = 0.99, 95% CI = 0.78–1.26, *P* = .94), and Weighted mode (OR = 0.97, 95% CI = 0.75–1.27, *P* = .85).

#### 3.2.4. Impact of gout on thyroid cancer

7 SNPs associated with gout were extracted as IVs. The results of MR Analysis show that, IVW (OR = 1.02, 95% CI = 0.89–1.17, *P* = .77), MR-Egger (OR = 1.02, 95% CI = 0.78–1.33, *P* = .88), Weighted median (OR = 1.02, 95% CI = 0.87–1.20, *P* = .76), and Weighted mode (OR = 1.02, 95% CI = 0.83–1.27, *P* = .83).

The results of this MR Showed that gout had no causal relationship with autoimmune hypothyroidism, autoimmune hyperthyroidism, thyroid cancer and thyroid nodule.

## 4. Discussion

To our knowledge, this is the first study to investigate the causal relationship between thyroid diseases (autoimmune hypothyroidism, autoimmune hyperthyroidism, thyroid nodules, and thyroid cancer) and gout. In this MR Study, we found that autoimmune hypothyroidism and autoimmune hyperthyroidism have positive causal effects on the risk of gout. It is suggested that autoimmune hypothyroidism and autoimmune hyperthyroidism play causal roles in the pathogenesis of gout. There was no causal effect of thyroid nodules and thyroid cancer on the risk of gout. In addition, gout has no causal effect on autoimmune hypothyroidism, autoimmune hyperthyroidism, thyroid nodules, and thyroid cancer.

Gout is a metabolic disease caused by high uric acid.^[20]^ The risk factors for gout are both non-genetic and genetic. Age, ethnicity, dietary habits and medications are key risk factors for the development of hyperuricemia and gout. Sodium salt is the main form of uric acid in human body. After sodium salt is deposited in joint cavity, soft tissue, cartilage and kidney through systemic circulation, sodium urate crystal is formed, and urate crystal can mediate gouty inflammation by activating phagocytic cells, inflammasome and toll-like receptors.^[21]^ Hyperuricemia is the key to the formation of gout, and is also a risk factor for metabolic diseases such as hypertension, type 2 diabetes, and cardiovascular disease.^[22,23]^ Gout is a polygenic genetic disease with complex genetic pattern and pathogenesis, involving multiple pathways and influenced by multiple environmental factors.^[24,25]^ Genome-wide association analysis (GWAS) identified many single nucleotide polymorphism loci associated with gout,^[26–28]^ it helps us to understand the genetic factors of gout from the genetic point of view and identify the risk genes of gout.

Many clinical studies have shown that there is a certain association between thyroid disease and gout. A study of 87,813 participants in Taiwan, China, showed that both hyperthyroidism and hypothyroidism were significantly associated with gout.^[11]^ Another study in China showed that hyperthyroidism is associated with hyperuricemia, which is more pronounced in male patients.^[29]^A retrospective cohort study in Korea also found an increased risk of gout attacks in patients with hyperthyroidism.^[30]^A retrospective study of 48,526 participants found that uric acid levels were linearly correlated with FT3 and FT4.^[31]^In addition, studies have found that the occurrence of hyperuricemia in hyperthyroidism and hypothyroidism patients is significantly increased. Among them, hyperuricemia in hyperthyroidism patients is caused by the increase of urate. Hyperuricemia in patients with hypothyroidism is caused by reduced renal blood flow and impaired glomerular filtration.^[32]^

At present, the specific mechanism of autoimmune hypothyroidism, autoimmune hyperthyroidism leading to gout has not been clarified. Purine and nucleotide metabolism is one of the causes of uric acid formation. Studies have shown that thyroid hormones can accelerate purine nucleotide metabolism.^[33]^Period-2 (Per2) is an important gene fou circadian clock.Per2 influences the transcription and expression of target genes by inhibiting the Bmal1-Clock complex, and expands its regulatory capacity by interacting with transcription factors such as nuclear receptors, which is the biochemical regulatory mechanism of Per2 on metabolism.^[34]^Studies have shown that T3 can affect the levels of enzymes involved in nucleotide metabolism in the liver and promote urate production by inducing Per2 expression, revealing a potential mechanism between thyroid disease and gout.^[35]^In addition, thyroid hormones can affect kidney development, kidney structure, and glomerular filtration rate. Hyperthyroidism and hypothyroidism are one of the key factors affecting renal function. Studies have found that thyroid hormones can affect kidney function, resulting in abnormal serum uric acid levels in patients.^[36]^Studies have shown that hyperuricemia in patients with hypothyroidism is associated with decreased uric acid clearance, and its specific mechanism is caused by decreased renal plasma flow and glomerular filtration rate (GFR).^[37]^Thyroid hormone may be involved in renal uric acid clearance. Reduced uric acid excretion and no increase in creatinine clearance in hyperthyroidism is the key to hyperuricemia.^[38]^

Epigenomics is the study of epigenetic modifications at the genomic level. Epigenetics regulates gene expression and has been shown to play a key role in a variety of metabolic diseases, such as gout, hyperthyroidism, and hypothyroidism.^[39]^ One study explored the enrichment of differential methylation in gout related adaptive immunity, including pathways for B and t cell receptor signaling, IL-17 signaling, and Th17 development.^[40]^Another study found 7 DNA methylation sites in gout patients that correspond to 7 genes, namely PGGT1B, UBAP1, RAPTOR, INSIG1, ANGPTL2, CNTN5, and JNK1.^[41]^ It has been found that the DNMT1rs228611 polymorphism may play a role in the development of increased gout in patients with gout.^[42]^ Graves’ disease is one of the causes of hyperthyroidism. It has been found that dysregulation of DNA methylation and histone modification of T cell signaling genes is involved in the development of GD. GD patients have low methylation and lower DNMT1 expression in B and T lymphocytes.^[43]^ In addition, DNA methylation, such as MAPK, Ras, and WNT, may be involved in associated hypothyroidism.^[44]^These studies suggest that epigenomics may be a key bridge in the study of the co-pathogenesis of gout and thyroid disease, the underlying mechanisms of which need to be further studied. p62,a 62kDa protein, was originally identified as a phosphotyrosine-independent ligand of the Srchomology 2 (SH2) domain of the lymphatic-specific Src family tyrosine kinase p56lck.^[45]^ Studies have shown that p62 is involved in human metabolic processes, such as lipogenesis,^[46]^ insulin signaling^[47]^ and inflammation.^[48]^ p62 plays a key role in the incidence of many metabolic diseases, including gout^[49]^ and thyroid diseases.^[50]^ p62 may be a potential mechanism for the causal association between gout and thyroid disease. The role of p62 in gout and thyroid disease needs further study. The hypothalamic-pituitary-thyroid axis plays an important role in regulating reproductive function. Abnormal thyroid function and thyroid diseases will adversely affect female reproductive function and lead to abnormal levels of female sex hormones, which will lead to irregular menstruation, infertility, poor pregnancy outcome and premature ovarian failure.^[51]^ Interestingly, studies have found that there are sex differences in the incidence of gout, and female estrogen and progesterone can reduce the risk of gout.^[52]^ It also provides some ideas for us to study the relationship between thyroid disease and gout.

We used a 2-sample MR Study to reveal the causal relationship between autoimmune hypothyroidism and autoimmune hyperthyroidism on gout, which is helpful for further research. Our research has the following advantages. First, to date, this is the first study using MR Methods to investigate the causal relationship between autoimmune hypothyroidism, autoimmune hyperthyroidism, thyroid nodules, and thyroid cancer and gout. Secondly, we used a rigorous IVs selection, multiple MR Methods and pleiotropic testing to ensure the reliability of the results. Finally, all included populations were European, which reduced confounding bias.

Of course, there are some limitations to this study. First, the study only included people of European descent and cannot prove whether there are genetic differences between different ethnic groups, countries and regions. Second, MR fails to further explore the biological mechanisms of autoimmune hypothyroidism, autoimmune hyperthyroidism, thyroid nodules, and thyroid cancer and gout.

## 5. Conclusions

Using 2-sample MR Analysis, we investigated the relationship between autoimmune hypothyroidism, autoimmune hyperthyroidism, thyroid nodules, thyroid cancer, and gout. We found a causal relationship between autoimmune hypothyroidism, autoimmune hyperthyroidism, and gout, all of which increase the risk of gout. There is no causal relationship between thyroid cancer, thyroid nodules and gout. There is no causal relationship between gout and autoimmune hypothyroidism, autoimmune hyperthyroidism, thyroid nodules, and thyroid cancer. In future studies, we need a larger sample size to verify the causal relationship and clarify the mechanism from multiple disciplines and perspectives.

## Acknowledgments

For the data used in these analyses, we gratefully acknowledge the ieu open gwas project (https://gwas.mrcieu.ac.uk).

## Author contributions

**Data curation:** Congcong Jia, ChunJing Li.

**Writing – original draft:** Shuai Song.

**Writing – review & editing:** Yuxia Ma.

## Supplementary Material
