# Supplementary material for: The causal association between thyroid disease and gout: A Mendelian randomization study
Source: Medicine (Baltimore). 2023 Nov 3;102(44):e35817. doi: 10.1097/MD.0000000000035817 (PMC10627627; doi:10.1097/MD.0000000000035817)
Supplement: Supplementary file 1 [file medi-102-e35817-s001.docx]

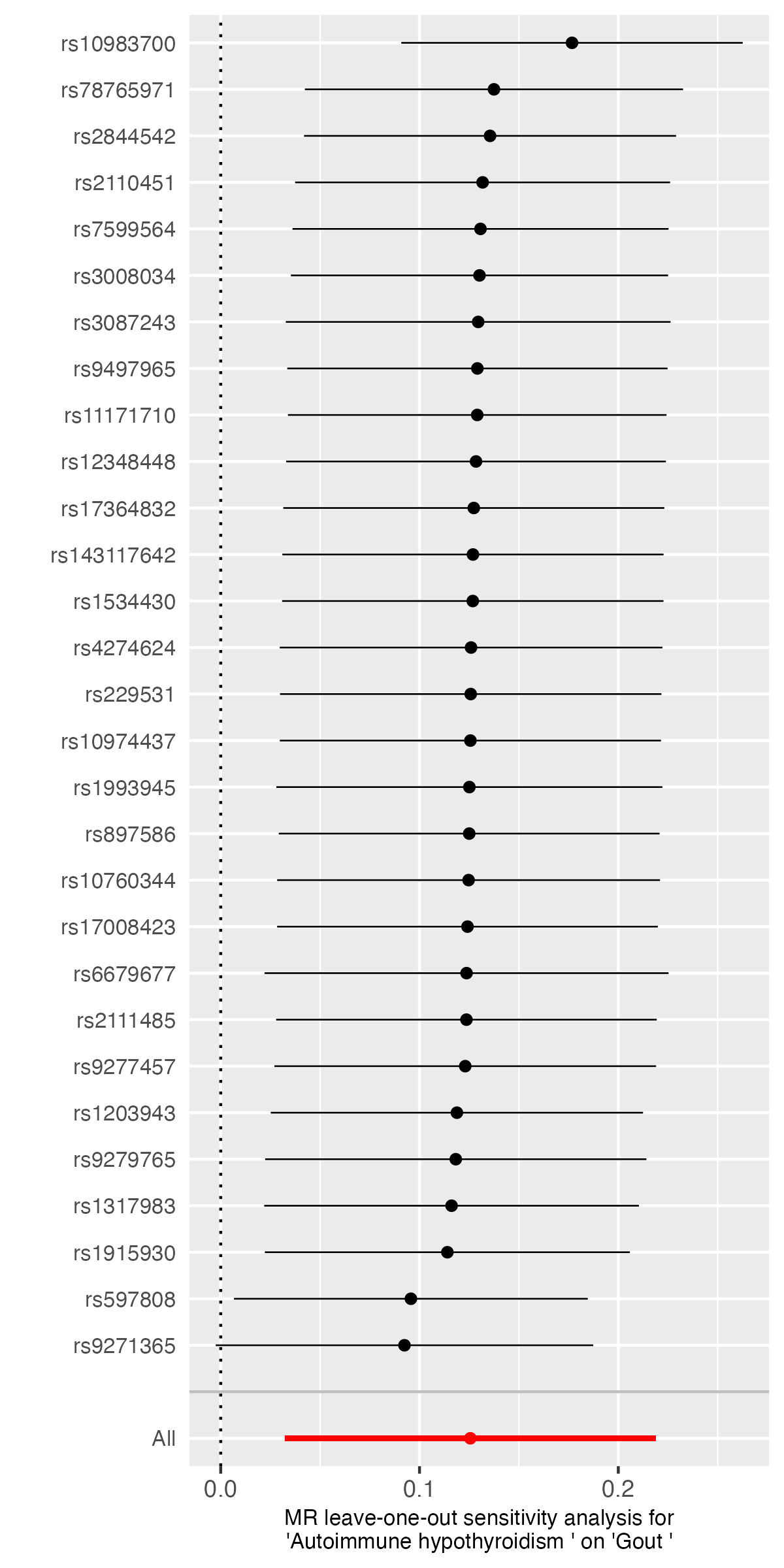

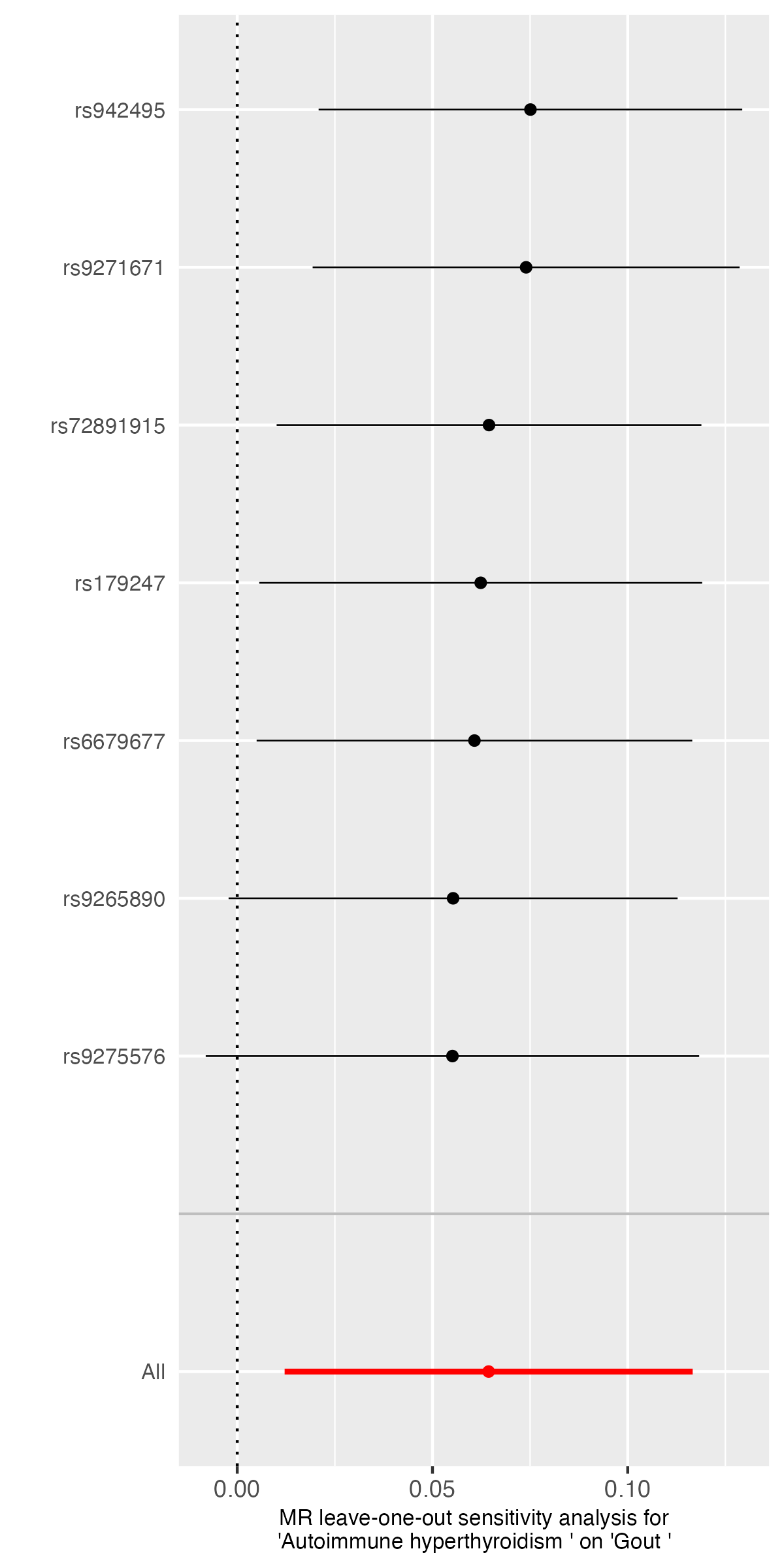


(Supplementary Figure 1A) (Supplementary Figure 1B)


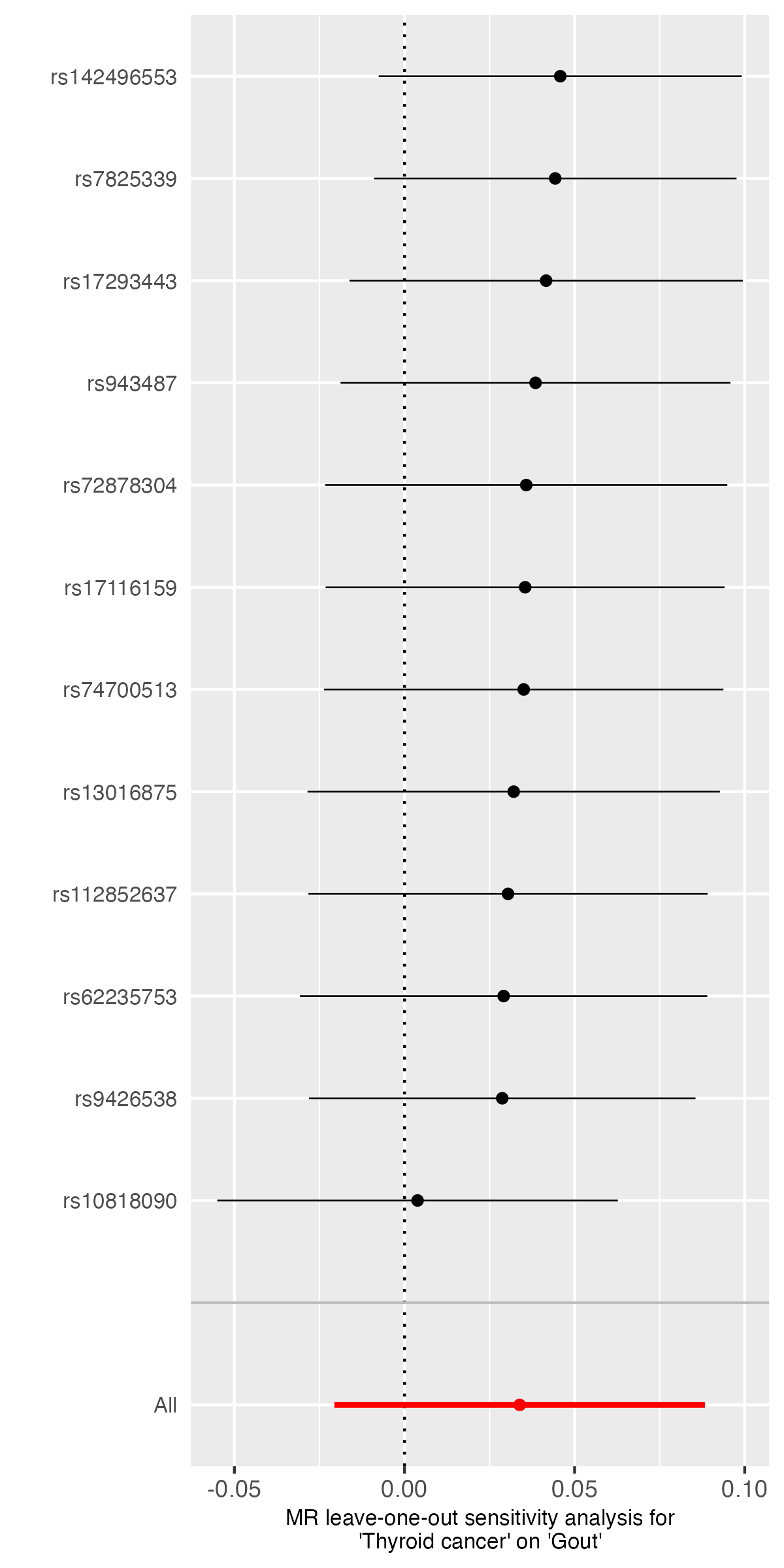

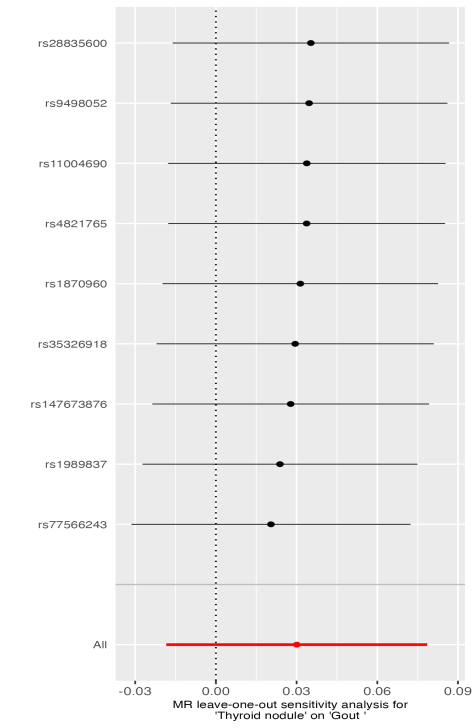


(Supplementary Figure 1C) (Supplementary Figure 1D)
